# Large melt diversity at a mid-ocean ridge thermal low

**DOI:** 10.1126/sciadv.adv4654

**Published:** 2025-04-30

**Authors:** Daniele Brunelli, Léna Verhoest, Marco Ligi, Christophe Hemond, Marcia Maia, Azam Soltanmohammadi, Federico Lugli, Philippe Nonnotte, Anna Cipriani

**Affiliations:** ^1^ Dipartimento di Scienze Chimiche e Geologiche, Università di Modena e Reggio Emilia, Modena, 41125, Italy.; ^2^ Woods Hole Oceanographic Institution, Woods Hole, 02543-1050 MA, USA.; ^3^ Istituto di Geologia Ambientale e Geoingegneria CNR, 00185 Roma, Italy.; ^4^ Geo-Ocean, Univ Brest, CNRS, Ifremer, UMR6538, F-29280 Plouzané, France.; ^5^ Istituto di Scienze Marine-CNR, 40129 Bologna, Italy.; ^6^ Natural Resources Canada, Geological Survey of Canada, Québec, G1K 9A9, Canada.; ^7^ Lamont Doherty Earth Observatory of Columbia University, Palisades, 10964 NY, USA.

## Abstract

Mid-ocean ridges serve as key sites for understanding the composition of the mantle, but extensive melting usually masks its lithological diversity. This study explores how cold mid-ocean ridge segments, such as the eastern Romanche ridge-transform intersection (ERRTI), provide unique insights into mantle heterogeneity. Here, a thick cold lithosphere faces the warm ridge segment efficiently cooling the ridge tip, thus reducing melting and mixing, and allowing distinct short-scale lithologies to be sampled. Our findings reveal a mosaic of mantle components with diverse geochemical and isotopic signatures, reflecting dynamic mantle processes over time. By examining these cold regimes, this research sheds light on the mantle’s compositional complexity and its evolution, offering a fresh perspective on lithospheric dynamics and melt generation in settings independent of hotspot influences.

## Teaser

Fine-scale mantle variability is unveiled at ridge thermal lows where melts from single heterogeneities are extracted separately.

## INTRODUCTION

**Fig. 1.**
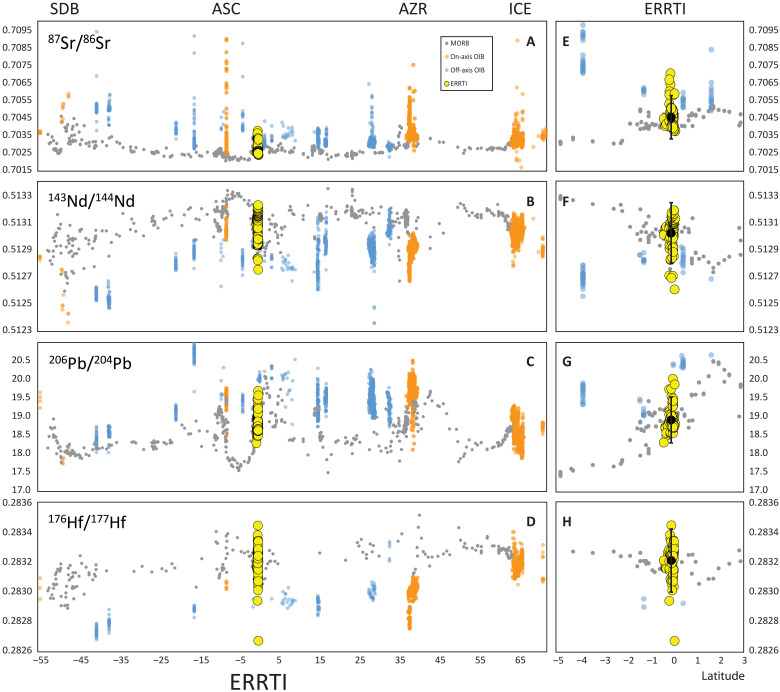
Isotopic variability of MORB glasses sampled along the entire MAR. (**A**) ^87^Sr/^86^Sr, (**B**) ^143^Nd/^144^Nd, (**C**) ^206^Pb/^204^Pb, and (**D**) ^176^Hf/^177^Hf versus latitude. Yellow large circles: MORB glasses from this study. Gray dots: MORB glasses downloaded from PetDB (https://earthchem.org/). On-axis hot spots are plotted as orange dots; blue dots are off-axis hot spots (data downloaded from Georoc and filtered for age <1 Ma, DIGIS Team, 2024, “GEOROC Compilation: Ocean Island Groups”, https://doi.org/10.25625/WFJZKY, GRO.data, V10; CC BY-SA 4.0, https://creativecommons.org/licenses/by-sa/4.0/). SDB, Shona-Discovery-Bouvet hotspot; ASC, Ascension; AZR, Azores; ICE, Iceland. (**E** to **H**) Enlarged view of the equatorial stretch across the sampled region. The average of all measured glasses plots on the regional trend.

Extensive evidence suggests that the Earth’s upper mantle comprises multiple chemically distinct components, particularly from an isotopic standpoint (*1*–*3*). At mid-ocean ridges (MOR), mantle heterogeneities are largely homogenized through melting processes, yielding basalt compositions that are biased toward the prevalent depleted mid-ocean ridge basalt (MORB) mantle (DMM) endmember (*3*–*5*). This homogenizing effect is especially apparent when considering the notable isotopic contrast between MORB and associated mantle rocks (*6*–*11*) as well as the inherent variability in incompletely aggregated gabbros (*12*) and melt inclusions (*13*–*17*). Globally, MORBs are characterized by depleted isotopic signatures, with more enriched signatures observed along MOR axes influenced by hot spots. Along the Mid-Atlantic Ridge (MAR), enriched isotopic signatures are particularly prominent in sectors near hot spots, such as Iceland, the Azores, Ascension, Discovery-Shona, and Bouvet (Fig. 1). Conversely, highly depleted signatures are observed at the Mohns and Knipovitch ridges and in the Lucky Strike region, reflecting ancient, depleted mantle domains preserved through time, despite the convection-driven stirring and progressive solid-state mixing that accompany mantle convection (*11*, *18*).

Large-scale isotopic variations along the MAR are generally attributed to interactions between the ridge and nearby hot spots (*19*–*21*). However, small-scale isotopic fluctuations within individual ridge segments remain less well understood and often are linked to localized heterogeneities within the mantle source (*22*, *23*).

MORB lavas are commonly considered a product of pooled partial melts that form at different depths and originate from distinct mantle reservoirs. Disentangling the specific contributions of each mantle heterogeneity to the extracted melt remains a challenge. At average mantle potential temperatures (1350°C), the subridge melting region is sufficiently large to induce extensive partial melting of the prevalent, and most refractory, DMM component (*10*, *24*). Any heterogeneity more fertile than the DMM dispersed in the source is diluted within this dominant DMM-derived melt, resulting in a chemically homogenized MORB mixture. The result is a chemically smooth MORB blend where enriched components only emerge sporadically or induce subtle local shifts in the isotopic signature when the degree of melting is reduced (*25*). The MORB blend defines the depleted boundary of the isotopic variability observed in a basin, as seen in Fig. 1 where MORBs consistently plot at the depleted end of the isotopic spectrum for both on- and off-axis hot spot variability. As mantle potential temperature (or spreading rate) decreases, the melting region shrinks, proportionally reducing the number of separated mantle components entering the melting region and sampled by partial melting (*4*, *10*, *26*). Under these conditions, the thermodynamic effect of heat diffusion into the low-solidus, first-melting lithology should enable the extraction of melts with reduced mixing or, in extreme cases, melts derived from individual components. This is because the lower solidus reservoir undergoes enhanced melting due to heat diffusion into the melting heterogeneity from the surrounding more refractory screen (*10*, *24*, *27*, *28*). Heat diffusion promotes overmelting of the low-solidus components while reducing the degree of melting of the more refractory components, regardless of the relative melt productivity of the two units (*10*, *24*, *29*).

**Fig. 2.**
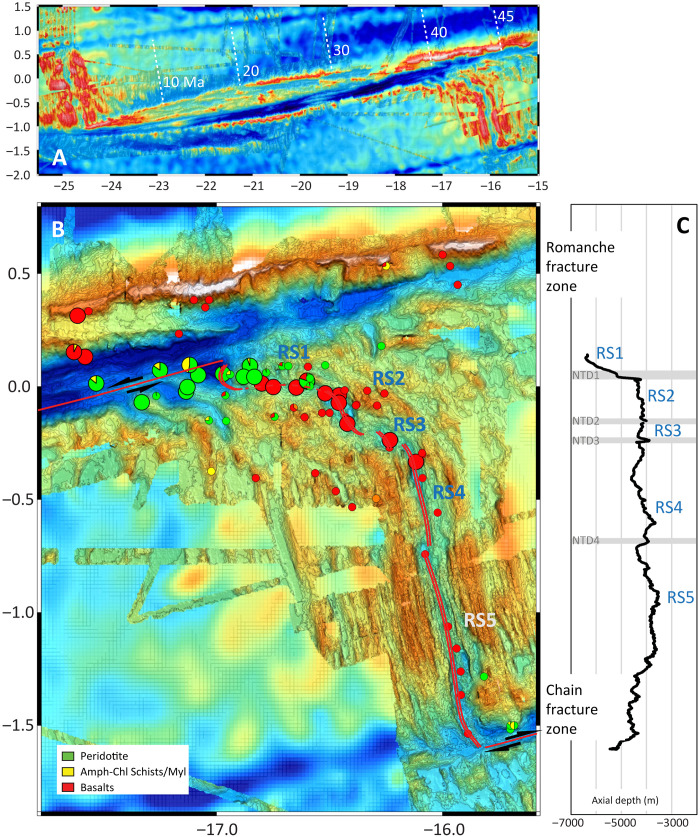
Bathymetric map of the Romanche-Chain ridge segment in the equatorial MAR. (**A**) Overview of the entire fracture zone, with the Romanche offsetting the MAR by ∼950 km and generating an age contrast of ∼45 Ma. (**B**) Shipborne bathymetry of the Romanche-Chain ridge segment. Four nontransform discontinuities (NTD1 to NTD4) divide the ridge into five subsegments named RS1 to RS5. Sample distribution is color coded by lithology. Large symbols are Nautile dives from the SMARTIES expedition in 2019, and small symbols are previous dredge stations (see the main text). The ERRTI region corresponds to the northern area from RS1 to RS3, characterized by oblique spreading and rough topography, which culminates in a magmatic spreading at the northernmost tip of the segment. (**C**) Along-axis bathymetric profile showing the positions of the nontransform discontinuities (NTD) and the ridge subsegments (RS).

We expect this effect to be amplified in ridge portions with lower-than-average temperatures, referred to here as “cold spots” (*30*, *31*). Cold spots have been identified in the context of oceanic mega-transforms (*30*, *32*), which not only create large offsets in the MOR system but also juxtapose oceanic lithosphere of different ages. As a consequence, older, thicker, and colder lithosphere encounters a warmer axial segment tip on both sides of the transform (Fig. 2), creating a strong lateral thermal gradient that progressively cools the mantle beneath the ridge axis (*32*–*34*).

The Romanche transform fault, the largest mega-transform along the MAR, offsets the ridge axis by ∼950 km, generating an age contrast of about 45 million years (Ma). The MAR segment comprised between the Romanche and the Chain fracture zones (Fig. 2) is characterized by extreme lateral thermal gradients (*34*). Its northern sector, hereafter called the eastern Romanche ridge-transform intersection (ERRTI) region, is characterized by rough topography and oblique spreading (Fig. 2). Here, the presence of a thermal minimum was first hypothesized on the basis of evidence of decreased degree of melting in mantle residua (*30*, *31*, *35*, *36*), peculiar RTI morphology (*33*), and rather extreme MORB compositions (*20*, *30*, *34*, *36*).

## RESULTS

### Lithological and chemical variability along the Romanche-Chain ridge

The Romanche-Chain ridge segment presents an asymmetric morphology with five subsegments separated by four nontransform discontinuities (Fig. 2). A well-developed ridge-parallel abyssal hill fabric characterizes the southern subsegment RS5 approaching the Chain Fracture Zone (FZ), suggesting that it is supported by a vigorous magmatism. Moving northward, the topography becomes progressively more irregular and rougher. Three discontinuities separate irregular basins, overall forming a large oblique structure accompanied by the increase in axial depth from RS4 to RS1. The subsegments RS2 and RS3 lay between 4200 and 4500 meters below sea level (m.b.s.l.), locally reaching 4800-m depth. Notably, the nodal basin at the termination of RS1 near the Romanche transform reaches a depth of 6920 m.b.s.l., making it the deepest nodal basin of the entire MAR (Fig. 2). These observations are indicative of a decreased magma supply toward the north as evidenced by the extensive outcropping of mantle rocks at the northern tip of the segment, where basalts have been only occasionally sampled (Fig. 2B) (*31*).

To characterize the compositional variability along the lateral thermal gradient at the ERRTI, high-resolution sampling has been carried with the manned submersible Nautile during the SMARTIES cruise (*37*), complementing and integrating existing on- and off-axis sampling by dredging (*30*, *38*–*40*).

**Fig. 3.**
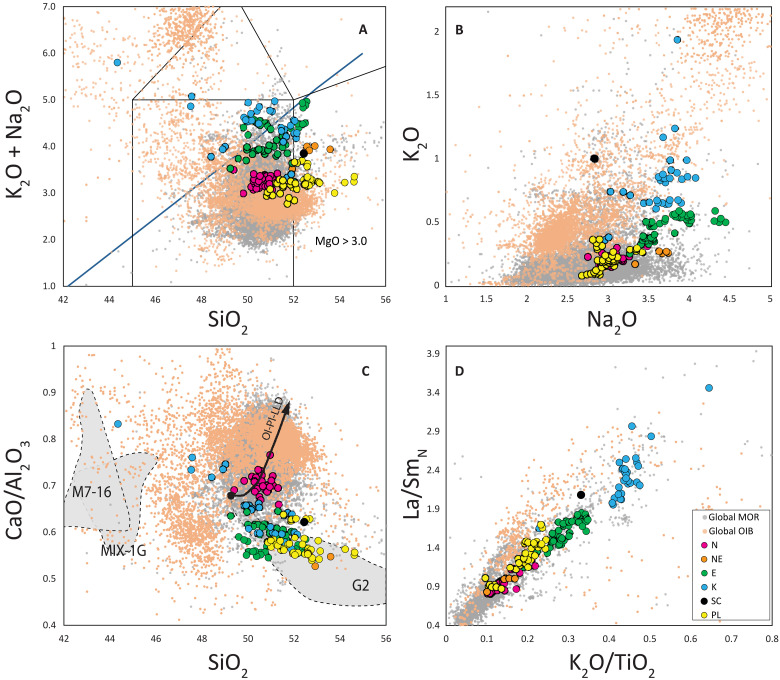
Composition of the ERRTI basaltic glasses. (**A**) Total alkali versus silica (wt %), (**B**) K_2_O versus Na_2_O (wt %), (**C**) CaO/Al_2_O_3_ versus SiO_2_ (wt %), and (**D**) La/Sm_PM_ [primitive mantle normalized to (*41*)] versus K_2_O/TiO_2_. Samples are color coded according to their isotopic, trace, and major element systematics: N, normal-MORB (magenta solid circles); NE, normal-enriched MORB (orange circles); E, E-MORB (green solid circles); K, K_2_O-rich MORBs (blue solid circles); SC, a compositionally unique MORB (black circles); PL, MORBs with plagioclase fingerprint (yellow circles). Small solid circles are respectively global MORB basaltic glasses (gray circles) and global OIB glasses (light orange circles). Both compilations consider basalts with MgO > 3.0 (wt %). G2 and M7-16 dashed gray fields represent the composition of experimental melts of anhydrous silica-enriched eclogite G2 after (*43*, *47*) and two silica-deficient pyroxenites: M7-16 (*84*) and MIX-1G (*54*). The arrow represents the olivine-plagioclase liquid line of descent from a primitive melt.

Major and trace elements (Fig. 3 and data S1) of the ERRTI basaltic glasses show a pronounced increase in alkali and incompatible elements as the samples approach the ERRTI: Na_2_O reaches up to 4.45 wt %, K_2_O up to 1.95 wt %, and La/Sm_PM_, normalized to (*41*), up to 3.6, expanding on observations from previous studies (*30*, *34*, *36*). Overall, the ERRTI MORBs fall at the alkali-rich end of the tholeiitic compositional field, encompassing both silica-saturated and silica-undersaturated terms, with the latter showing strong alkali enrichment, even extending into the alkali basalt field (Fig. 3A). Notably, K_2_O shows a marked enrichment alongside Na_2_O, reaching up to 1.95 wt % (Fig. 3B), a rare feature among MORBs far from hotspots. This K_2_O enrichment is a distinctive mark of the ERRTI “alkaline MORBs” that cross the alkaline-tholeiitic boundary in the Total Alkali versus Silica (TAS) classification (Fig. 3A). These samples are nepheline normative (0.3 to 16.5%; fig S1) and have high K_2_O/TiO_2_ and La/Sm_PM_ (Fig. 3D).

**Fig. 4.**
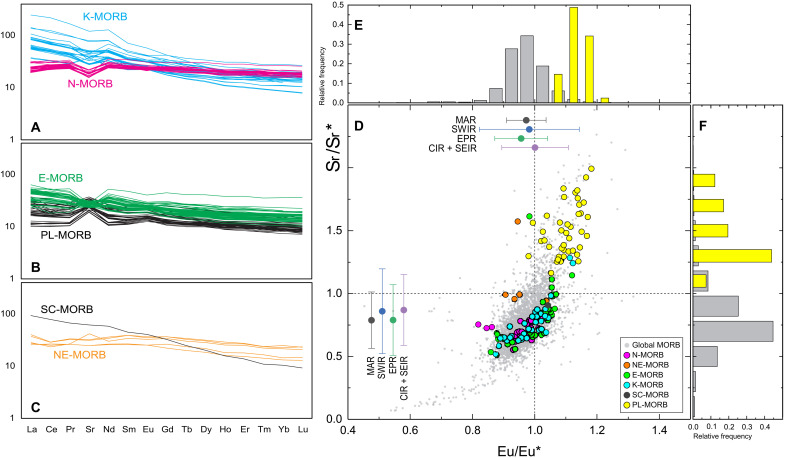
REE + Sr patterns of the six compositional groups at the ERRTI. (**A** to **C**) Patterns of K- and N-MORBs, E- and PL-MORBs, and SC- and NE-MORBs, respectively. (**D**) Strontium anomaly versus europium anomaly of the ERRTI samples compared with global MORB distribution. Averages of single ridge segments are plotted on the relative axis. EPR, East Pacific Rise; CIR and SEIR, Central and Southeast Indian Ridges, respectively. Sr and Eu anomalies are calculated as (*Sr*_*N*_/Sr*) and (*Eu*_*N*_/Eu*) respectively, where Sr* = (*Ce*_*N*_ *× Nd*_*N*_)^1/2^ and Eu* = (*Sm*_*N*_ *× Gd*_*N*_)^1/2^. (**E** and **F**) Frequency plots showing the relative distribution of the ERRTI PL-MORBs with respect to the global MORB distribution.

A peculiarity of the lavas sampled in this region is the high variability of CaO/Al_2_O_3._ While the CaO/Al_2_O_3_-positive trends in N-MORBs and tholeiitic ocean island basalts (OIBs) can result from fractional crystallization of plagioclase from a peridotitic derived melt (Fig. 3C), the ERRTI samples exhibit a broad range from very low to high CaO/Al_2_O_3_, forming a distinctive negative trend with SiO_2_ (Fig. 3C). Low CaO/Al_2_O_3_ ratios have also been documented in MORBs from the western Southwest Indian Ridge (SWIR), another cold sector of the MOR, and attributed to melting of a Cpx-depleted source (*42*). Minor and trace elements, such as K_2_O/TiO_2_ and La/Sm_PM_ ratios, also show considerable variability, largely encompassing the MORB field (Figs. 3 and 4). Rare earth elements (REEs) range from normal to enriched terms, with most samples showing marked enrichment; strongly depleted compositions are notably absent (Fig. 4).

**Fig. 5.**
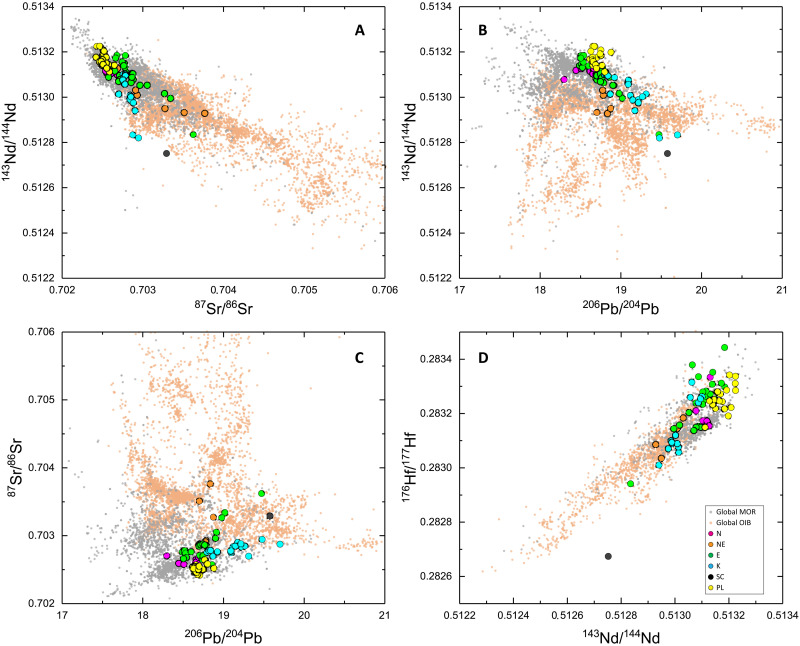
Isotopic composition of the ERRTI basaltic glasses compared to global MORB and OIB distribution. (**A**) Nd versus Sr, (**B**) Nd versus Pb, (**C**) Sr versus Pb, and (**D**) Hf versus Nd isotopic compositional fields. The variability observed at the ERRTI encompasses the global MORB variability and spans a large part of the global OIB range (unfiltered dataset from DIGIS Team, 2024, "GEOROC Compilation: Ocean Island Groups", https://doi.org/10.25625/WFJZKY, GRO.data, V10; CC BY-SA 4.0, https://creativecommons.org/licenses/by-sa/4.0/).

The isotope variability of the Romanche-Chain ridge basalts extends beyond the typical MORB field, spanning a large portion of the global OIB field (Fig. 5). The Sr, Nd, Pb, and Hf isotope compositions of the ERRTI basaltic glasses vary widely, from ultradepleted to highly enriched values (Fig. 5). Specifically, the ^87^Sr/^86^Sr ratios range from 0.702398 to 0.703758; ^143^Nd/^144^Nd vary from 0.512752 to 0.513225 (ɛNd from 2.3 to 11.5) and ^206^Pb/^204^Pb from 18.503 to 19.700. The ^176^Hf/^177^Hf ratios vary from 0.282674 to 0.283443 (ɛHf from −2.9 to 23.7), a range that covers most of the isotope variability observed across the entire MAR system and the global OIB field (Figs. 1 and 5D; −2.76 at 47.3°S and 26.21 at 40.2°N). This extensive isotope variability in the ERRTI basalts is particularly remarkable given the limited size of the sampled area, within segments RS1 and RS2, where the highest variability occurs, measuring only 20 and 35 km in length, respectively. The range of isotopic compositions at the ERRTI suggests highly efficient separation of melts from diverse source reservoirs during the subridge partial melting and the absence of homogenization in shallow magma chambers and during transport. This makes the ERRTI an ideal setting for isolating and identifying distinct mantle components.

### Definition of the compositional groups

The analyzed basaltic glasses have been classified into four major compositional groups: normal (N-MORB), enriched (E-MORB), high-K (K-MORB), and ghost-plagioclase (PL-MORB), along with two minor groups: normal-enriched (NE-MORB) and subcontinental (SC-MORB) (Fig. 3). These groups are not entirely distinct across all compositional spaces, suggesting mixing between components that creates overlapping geochemical characteristics. Hereafter, we define as N-MORB the basalts with low alkali content and high CaO/Al_2_O_3_ ratio; they exhibit REE patterns that parallel those defined in (*43*). E-MORBs have a markedly lower CaO/Al_2_O_3_ ratio than N-MORBs but similarly REE patterns paralleling those defined in (*43*). K-MORBs present the strongest REE enrichment with steep patterns typical of alkaline lavas (Figs. 3D and 4A). They exhibit a strong enrichment in K_2_O, reaching up to 2 wt % and form a compositional trend from high silica/low CaO/Al_2_O_3_ to low silica/high CaO/Al_2_O_3_ (Fig. 3C) and are generally Ne normative (fig. S1). With higher K_2_O at given TiO_2_ content than E-MORB (Fig. 3D and fig. S2A), K-MORBs resemble high-K basalt and absarokite within the shoshonite series as per Ewart’s classification (fig. S2B) (*44*). Considering all typical characters, K-MORB can be defined as alkaline-MORB: They encompass the McDonald-Katsura line in the TAS (Fig. 3A), have a steeply enriched REE pattern (Fig. 4A), reach the absarokite field in the K-Si diagram, and are nepheline normative reaching the basanite field (fig. S1). When examining the isotopic ratios (table S2), N-MORBs plot at the depleted end, while E-MORB and K-MORB follow diverging trends pointing to enriched EMII-type and HIMU-type terms, respectively (Fig. 5). These latter two groups show enrichments that extend beyond the MORB field, aligning closely with the range observed in global OIB variations (Fig. 5).

The three other compositional groups present characters distinct from the previous:

PL-MORBs lavas have similar alkali content to N-MORBs but range to higher SiO_2_ values and span the low CaO/Al_2_O_3_ field as E-and K-MORBs (Fig. 3). Their absolute REE content is generally lower than other lava types while showing mildly enriched patterns (Fig. 4). Their radiogenic isotope ratios plot at the most depleted end of local and global MORB trends (Fig. 5). They feature coupled Eu-Sr anomalies with exceptionally high values, among the most extreme along MORs (Fig. 4, B and D), statistically beyond the upper end of the global distribution of both Sr and Eu anomalies (Fig. 4, E and F).

NE-MORBs are compositionally intermediate between N- and E-MORBs. They have higher Na and Si and markedly lower CaO/Al_2_O_3_ than N-MORBs (Fig. 3C) and weakly convex upward and slightly enriched REE patterns (Fig. 4C). Their isotopic composition forms a separate trend pointing toward EMII-type compositions (Fig. 5C), diverging clearly from the trends of N- and E-MORB.

SC-MORB is represented by a single sample (SMA1966-128), which exhibits the highest enrichment in all isotopic systematics (Fig. 5) and the steeper REE pattern (Fig. 4C). The Hf isotopic composition of this sample is unusually low (^176^Hf/^177^Hf = 0.28674; εHf = −3.45), plotting well below the global MOR and OIB trend (Fig. 5D). Although it has a high K content (K_2_O = 1.01; Fig. 3B and fig. S2), its other compositional characteristics are intermediate between the E- and K-MORB groups (Figs. 3 and 5).

Overall, the isotope signatures and trace element patterns reflect substantial compositional variability in the source, suggesting that melts generated by distinct reservoirs within the melting region underwent limited mixing before eruption. This incomplete homogenization allows for the preservation of distinct source geochemical signatures, providing insight into the varied origins of MORB compositions within the Romanche-Chain ridge segment.

## DISCUSSION

### Along-axis thermal gradient: Effects of the enhanced cold edge effect

**Fig. 6.**
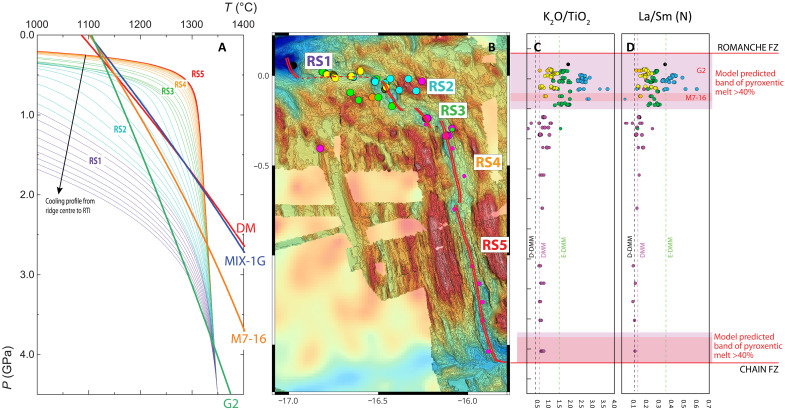
Along-axis distribution of the compositional groups and associated chemical variability compared with calculated thermal paths along the ridge. (**A**) Summary of thermal PT paths calculated at different ridge locations from the center of the warmer segment (RS5, red lines) to the RTI (RS1, violet lines). The solidi of the pyroxenites used in this paper are also reported along with the DM one. (**B**) Geographical distribution of the sampled MORBs color coded by compositional groups (same as previous figures). (**C**) K_2_O/TiO_2_ ratio and (**D**) La/Sm in MORB glasses normalized to primitive mantle of (*41*). The maximum enrichment appears in the northernmost 40 km of the axis (RS1 to RS2) where compositions range above the MORB field. A comparison with the D-DMM, DMM, and E-DMM compositions as defined in (*43*) shows the strong deviation approaching the northern RTI. Large symbols are data from this work, and small symbols are literature data color coded according to our compositional grouping. Light-red fields mark the model predicted bands where extracted melts are composed of a mixture containing more than 40% of pyroxenitic-derived melt (G2 and M7-16 are representatives of Si-rich and Si-poor members).

The lateral distribution of the observed compositional variability shows that the strongest enrichments are confined to the region close to the northern RTI (Fig. 6). Incompatible elements and isotopic enrichments are concentrated in the northernmost 40 km of the ridge axis, where the Romanche cold edge effect is expected to be most intense (Fig. 6). In contrast, “Normal” MORB values are observed in the central and southern sections of the ridge segment, along segments RS4 and RS5, where a well-developed abyssal hills seafloor fabric reflects abundant magmatic supply. Some previously published data for the Romanche-Chain ridge (*20*, *30*) have been classified according to the compositional groups discussed and are displayed in Fig. 6.

At the RTI, where the ridge intersects the transform, a temperature decrease occurs because of heat diffusion from the warmer ridge segment toward the cooler, older lithosphere adjacent to the RTI (*45*). Early modeling of the Romanche ERRTI cold edge effect demonstrated that this temperature drop results in increased concentration of volatiles and incompatible elements in the erupted melts while approaching the Romanche transform (*34*). However, the observed isotopic variability reported here cannot be accounted for solely by partial melting of a homogeneous mantle source, suggesting instead the presence of mantle source heterogeneities that contribute to the major and trace element, as well as isotopic, variability (Fig. 6).

Hereafter, we explore the hypothesis that the temperature decrease induced by the Romanche cold edge effect may cause low-solidus and high-solidus mantle components to melt separately during partial melting of a composite mantle beneath the Romanche-Chain ridge. Specifically, we examine the impact of varying proportions of low-solidus components within a DMM-type matrix. For the purposes of this study, we focus on binary source mixing between a fertile mantle component and a single low-solidus component.

The temperature field of the upper 150 km of the mantle has been derived using an isoviscous, incompressible mantle flow model within a half-space framework, driven by the divergence of rigid overlying plates, moving apart at a rate of 14.6 mm/year (*46*). This model simulates the equatorial MAR, capturing the configuration of major plate boundaries, including the long-offset Chain, Romanche, and St. Paul transform faults (fig. S3). Further methodological details are reported in the numerical and analytical methods subsection, “3D mantle thermal model.”

**Fig. 7.**
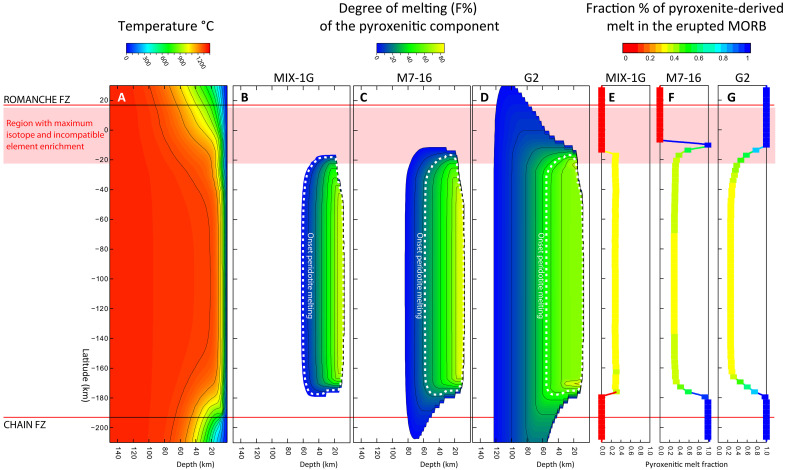
Results of MeltPX numerical experiments simulating partial melting of a mixed mantle source containing 6% of different pyroxenite types. (**A**) Thermal field (see Materials and Methods) with mantle Tp = 1305. (**B** to **D**) Spatial distribution of melting for MIX-1G, M7-16, and G2 pyroxenites, respectively, color coded by degree of melting. The white dashed line marks the onset of the host peridotite melting. (**E** to **G**) Estimated contribution of pyroxenite-derived melts to surface magmatism. At the outer limits of the melting region, only low-solidus lithologies melt, allowing for the extraction of melts derived exclusively from pyroxenites.

The thermal field beneath the ridge axis has been obtained by extracting two-dimensional (2D) thermal profiles at 3-km intervals along the Romanche-Chain ridge axis (Figs. 6 and 7A). As isotherms approach the northern and southern RTIs, they bend downward due to heat diffusion from the warm ridge sector toward the adjacent colder, older lithosphere (Fig. 7A). The asymmetry between the two RTIs is due to the age contrast and the resulting lithospheric thickness at the Romanche and Chain fracture zones, which result from different cooling times: about 14 Myr for the Chain FZ generating a 37-km-thick lithosphere versus ∼45 Myr for the Romanche FZ where the lithosphere thickens to 67 km (*34*, *46*). Using the MeltPX algorithm (*29*), we calculated the degree of melting and the relative amounts of melt produced from peridotite and pyroxenite along each thermal profile, assuming that melt generation occurs within isolated vertical columns without lateral transport (Fig. 7 and fig. S5). MeltPX allows differentiation of the melt contributions from a low-solidus component (modeled as pyroxenite) from that of the host peridotitic mantle. We tested three low-solidus compositions to explore different extents of thermal and chemical interaction during melting: G2, M7-16, and MIX-1G (*29*, *47*, *48*). They represent distinct pyroxenite types with varying solidi: silica-enriched eclogite (G2), silica-deficient/low-alkali/low-Mg# pyroxenite (M7-16), and a silica-deficient/high alkali/high Mg# pyroxenite (MIX-1G). Together, their solidi bracket the variability of experimental and natural pyroxenitic components (Fig. 6A). As peridotitic source, we used the fertile lherzolite of (*29*).

**Fig. 8.**
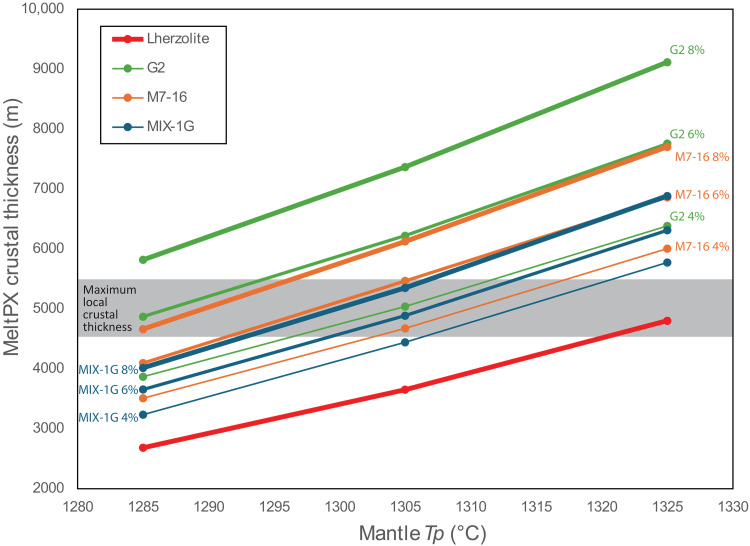
Results of MeltPX numerical experiments simulating partial melting of a mixed mantle source. Variable pyroxenite proportions and mantle potential temperatures have been used to calculate the total amount of melt produced, expressed as cumulative crustal thickness. Three pyroxenites types are tested: G2 (green lines), M7-16 (orange), and MIX-1G (blue) with increasing line thickness representing pyroxenite volumes of 4, 6, and 8%. The red curve represents the crustal thickness produced by melting a homogeneous lherzolite at the same mantle temperatures. The addition of pyroxenite into the mantle enhances melt production, depending on the relative solidus positions of the host lherzolite and the pyroxenite (see Fig. 6A). The gray band indicates the maximum local crustal thickness (5000 ± 500 m) from (*53*).

The overall extent of melting for the peridotite-pyroxenite mixture and thus the total melt volume and composition are influenced by mantle potential Temperature *Tp*, the relative amount of high- and low-solidus components, and the fertility contrast between them, i.e., the relative solidus position of each component (*10*, *24*, *29*). A series of numerical experiments reveals the influence of each parameter, as summarized in Fig. 8, fig. S5, and the Supplementary Materials. Increasing the amount of pyroxenite raises the total melt production (and hence crustal thickness; Fig. 8 and fig. S5), similar to the effect of increasing mantle potential temperature (*29*). We used the estimated maximum crustal thickness as an external constraint to define plausible values for mantle Tp and the maximum pyroxenitic content (Fig. 8). In segment RS5, located outside the influence of the cold edge effect, the estimated crustal thickness is approximately 5 km (*49*). This value can be achieved by melting a mantle mixture containing 4 to 8% of low-solidus components at a Tp of 1305°C (Fig. 8).

It appears that low contents of pyroxenitic materials dispersed within the subridge mantle can account for the observed along-ridge compositional pattern, influenced by varying combinations of temperature and pyroxenite content. Hereafter, we explore the thermal effects assuming a mantle Tp = 1305 and a homogeneous mantle composition along the ridge, with a constant 6% low-solidus component present in every section. This Tp is close to the lower end of MORB mantle temperatures estimated varying between 1280° and 1400°C, as reported in (*50*), consistent with the presence of a thermal low in the equatorial MAR (*30*). Numerical results are shown in Fig. 7 and fig. S5. Pyroxenite begins to melt before the host peridotite, as indicated by the white dashed line in Fig. 7 (B to D). Consequently, the melting region for pyroxenite extends further than that for peridotite (Fig. 7). The depth at which melting initiates depends on the relative fertility of each domain: MIX-1G exhibits the smallest melting region, while G2 the largest (Fig. 7, B to D). The heat subtracted during pyroxenite melting delays the onset of the peridotite melting, in proportion to the amount and fertility of the pyroxenite component (*10*, *29*). This results in a final melt composition that reflects the combined productivity of partial melting of both pyroxenite and peridotite. In the central part of the ridge segment, where the degree of melting is maximum, the cumulative crustal thicknesses produced by partial melting are 4.6, 5.0, and 4.6 km with 6% of MIX-1G, M7-16, and G2, respectively. The extracted melt contains 28.1, 29.7, and 25.4% pyroxenitic-derived melt, respectively. However, the situation changes near the lateral edges of the melting region. Here, the melting region of pyroxenite extends beyond that of peridotite (Fig. 7, B to D). In these peripheral zones, melts can be almost entirely derived from the low-solidus mantle component, as shown by the compositional profiles in Fig. 7 (E and F). This pattern results from the cooler thermal conditions at the ridge boundaries, where thermal paths cross the lower solidus of pyroxenite but not the higher solidus of lherzolite (Fig. 6A). Therefore, lateral cooling generates a region where only low-solidus lithologies can melt. The lateral extent of this region depends on the relative fertility of the low-solidus component (maximum for G2, smaller for M7-16, and negligible for MIX-1G; Fig. 7, B to D) and the intensity of lateral cooling, i.e., from the age contrast of the fracture zone, resulting in a more pronounced lateral extension to the south because of the lower age contrast of the Chain FZ with respect to the Romanche.

Pyroxenites with solidus between M7-16 and G2 have the potential to produce melts with a high pyroxenitic-derived component (>40%), reaching up to 100% approaching the FZ (Fig. 7, F and G). The model predicts the potential for extracting nearly pure pyroxenitic melts within the northernmost 40 km of the ridge, approaching the northern RTI. When the pyroxenite solidus is close to that of the peridotite, as in MIX-1G, the melting region does not sensibly extend (Fig. 7B), although melt productivity increases (Fig. 8), and the final MORB composition reflects the mixture of both components (Fig. 7E).

While this model explains the northern setting, it fails to explain the absence of enriched MORBs approaching the southern RTI toward the Chain FZ, despite substantial edge cooling (Figs. 6 and 7). Only a few samples have been analyzed in the southern cold edge region (Fig. 6) hinting at a possible sampling bias. However, it is important to note that the southern ridge segment (RS5) is sustained by strong magmatism and that the Chain FZ itself has a relatively small age contrast compared to Romanche (∼14 Ma). The cold edge effect is not prominent even in larger FZs, such as the Vema FZ at 11°N along the MAR, which has an age contrast of about 22 Myr (*9*, *10*, *51*). This may suggest that in magma-rich segments, melts extruded at RTIs are delivered laterally toward the RTI from the central part of the ridge via diking (*52*, *53*). Differences in seafloor morphology between the northern and southern RTIs (oblique spreading versus normal J-shaped abyssal hills) and in the lithological distribution (exhumed mantle versus diffuse basalt extrusion; Fig. 2) suggest a strong difference in the amount of magma delivered to these regions and a possible role for lateral transport in the southern RTI.

### Nature and composition of the endmembers

Numerical experiments presented in the previous section demonstrate that the enriched MORBs sampled in the ERRTI region likely represent melts derived predominantly from pure pyroxenitic sources. The geographic distribution of the enriched samples aligns with predictions from the thermal model, supporting the idea that thermal separation of low-solidus components is the primary mechanism driving the observed compositional distribution. This indirectly confirms that the geochemical anomalies arise from discrete lithologies with solidus temperatures lower than those of the host DMM peridotite (*10*, *24*, *25*).

This finding opens intriguing possibilities for exploring the intrinsic lithological heterogeneity of the local mantle source. However, it is notable that both the enriched samples (NE, E, K, and SC) and the relatively depleted PL group mainly plot at CaO/Al_2_O_3_ ratios lower than the typical MORB field and close to the experimental eclogite field represented here by the G2 experimental results (Fig. 3C). Together, they form a trend toward the silica-poor, high-CaO/Al_2_O_3_ values of the most enriched K-group samples. Low CaO/Al_2_O_3_ ratios are indicative of low-pressure melting of silica-enriched garnet-bearing pyroxenite (*47*, *54*), whereas partial melting of silica-deficient pyroxenites, represented by the compositional fields of MIX-1G and M7-16 in Fig 3C, produces melts with high CaO/Al_2_O_3_ and low silica content (*29*, *48*). The enriched-low CaO/Al_2_O_3_ trend in Fig. 3C can thus be interpreted as partial melting of silica-rich (G2-type) low-solidus/garnet-bearing components dispersed within a spinel-equilibrated DMM (N-MORB), with minor contributions from silica-deficient (M7-16–type) heterogeneities.

The extreme variability of naturally occurring pyroxenites complicates direct correlations between isotopic fingerprints and specific mantle lithologies. Moreover, mixing variable amounts of partial or incompletely aggregated melts can result in substantial isotopic dispersion, even when considering only two mantle endmembers. This dispersion arises from mixing of partial melts extracted from different depths (*5*, *55*) and/or incomplete aggregation of these melts (*56*). At the ERRTI, all enriched compositional groups are erupted in a restricted area (Fig. 6), suggesting that mixing among several endmembers may have occurred, even if the observed trends exhibit an apparent binary character. While a comprehensive analysis of the petrologic processes in the ERRTI mantle is beyond the scope of this paper, we focus here on the unique possibility that cold spots provide for observing mantle source variability, in contrast to hot spots. Here, we observe that (i) the MeltPX model predicts that in the ERRTI region, melts are dominated (up to 100%) by low-solidus components; (ii) in isotopic space (Fig. 5), each compositional group forms relatively coherent trends, suggesting that the most enriched samples are possibly formed from the mixing of large amounts of low-solidus–derived melts with smaller or negligible contribution from high-solidus DMM components, as predicted in Fig. 7 (E to G). To further investigate the composition of the enriched groups (E, K, and NE), we use simple binary mixing models.

**Fig. 9.**
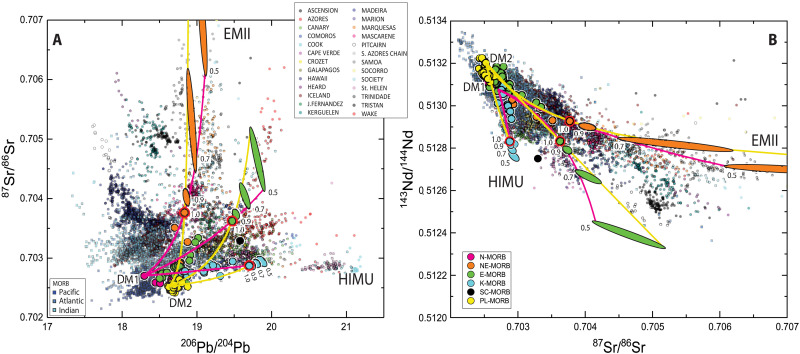
Composition of calculated conjugate endmembers for the K, E, and NE trends. Panels show (**A**) Sr-Pb and (**B**) Nd-Sr isotopic spaces and ellipses of predicted conjugate enriched source composition and relative mixing lines, which are color coded according to the depleted endmember. Each ellipse outlines the envelop of possible predicted enriched source compositions for the K, E, and NE groups. Calculations assume that the most enriched sample for each group (highlighted with a bold red rim) is either a pure low-solidus–derived melt (labeled 1.0) or a mixture of an unknown, more enriched conjugate component with two depleted endmembers. The two depleted components, DM1 and DM2, correspond to sample S1639-2 (N-MORB) and SMA1975-302 (PL-MORB), respectively. Ellipses are labeled according to the assumed amount of conjugate enriched endmember (0.9, 0.7, and 0.5 relative low-solidus content) in the mixture. MOR ridge basalts are represented by small squares and OIB by small circles (see legend in the figure).

The data highlight substantial variability within the depleted endmembers, suggesting fine-scale heterogeneity within the DM reservoir. To capture this complexity, we define two distinct depleted components, corresponding to N- and PL-MORBs, referred to here as DM1 and DM2, respectively. These components represent two separate depleted reservoirs with different geochemical and isotopic characteristics. To further characterize the enriched groups (K, E, and NE), we back-calculate from simple mixing equations the predicted enriched, low-solidus conjugate endmember for each trend. For this, we assume that our most enriched samples are either pure low-solidus melts or consist of 50 to 90% low-solidus–derived melts (Fig. 9). Calculations for each group yield a range of enriched compositions, represented as ellipses in Fig. 9, which illustrate the possible compositional variability of the enriched conjugate components. The lower the proportion of the enriched component mixed into the measured DM endmembers, the farther the possible primary enriched melts plot from them (Fig. 9).

The K-MORB conjugate endmembers plot close to the most enriched K sample (S1311-03), suggesting that this composition possibly represents a near pure low-solidus–derived melt. This group exhibits a HIMU-like signature (*2*, *21*, *57*), consistent with the field of the Madeira Island lavas and pointing toward the isotopic domain of St. Helen (Fig. 9A). The overlap with the HIMU field in Nd-Sr isotopic space (Fig. 9B) reinforces this connection, indicating a source enriched in radiogenic Pb and other HIMU-defining isotopic traits.

The most enriched E-MORB sample (SMA1973-246) may similarly represent a quasi-pure melt derived from a low-solidus lithology, with its conjugate endmembers plotting in an isotopically distinct region that is not fully covered by the global MORB or OIB field. This trend partially aligns with isotopic arrays of the Azores and Trinidade-Vitoria, and it closely follows the Comoros and Galapagos trends and clearly points to the Pitcairn trend in the Nd-Sr isotopic space, although it diverges in the Sr-Pb space. Overall, this trend converges toward the isotopic region defined as Focal Zone (FOZO) in (*58*), a common proposed prevalent plume component with intermediate or depleted Sr, Nd, and Pb isotopic compositions, to which MORBs appear to gravitate.

The NE conjugate endmembers, in contrast, plot farther from the most enriched ERRTI samples, suggesting a more diluted low-solidus–derived component. This trend aligns with the Samoa-Society-Marquesas paths in all compositional spaces, pointing to the EMII component (*2*, *21*).

The two depleted poles, N- and PL-MORBs, have two distinct characters. N-MORBs entirely match the isotopic, major, and trace element compositions of N-MORBs as defined in (*43*) representing the DMM source that dominates MORB chemistry worldwide. By converse, the PL-MORBs show geochemical features indicative of Eu and Sr enrichments, highly isotopically depleted materials in the local subridge mantle source. This geochemical fingerprint, recognized in both basalts and melt inclusions from various segments of the MOR (*59*–*62*), is often defined as “ghost plagioclase” (*63*). The isotopically depleted ratios recorded in these basalts rule out recent assimilation during melt percolation through crystal mush cumulates or formed cumulates in the oceanic lithosphere (*61*, *62*). Their high Al contents and depleted isotope ratios also exclude the presence of kinetic effects generating coupled Eu-Sr–positive anomalies during spinel-field partial melting of peridotite due to faster diffusivities of Eu and Sr than neighboring elements in clino- and orthopyroxene (*60*, *64*). Instead, we argue that partial melting of a recycled meta-gabbroic pyroxenite mixed with peridotite-derived melts provides a better explanation, as shown in (*59*).

The single basaltic glass in the SC group cannot confidently be used to derive a conjugate endmember. While it shares some compositional traits with both K-MORB and E-MORB, it is unique in showing an exceptionally low Hf isotopic ratio (εHf = −3.45). To our knowledge, oceanic basalts with such a low εHf for a given εNd have not been previously documented. This unusual isotopic signature can be modeled by mixing 50 to 70% of a 2–billion year (Ga)–old recycled oceanic crust (*65*) with a DMM source. However, similar isotopic signatures have been reported in kimberlites from South Africa (*66*) and, more pertinently, in the nearby Sierra Leone continental margin (*67*). The latter features isotopic ages compatible with the opening of the equatorial Atlantic, suggesting that this distinct sample, recovered in a highly tectonized region at the extremity of the ridge segment (Fig. 6), may represent a remnant of the pre-oceanic crust. Such remnants are known to become trapped by oscillatory spreading as shown by sedimentary rocks older than the ages predicted by normal seafloor spreading (*68*, *69*). On the basis of these characteristics, we tentatively suggest that the SC sample may have sampled a fossil fragment of subcontinental lithospheric mantle.

### The ERRTI mantle source assemblage

Melting of the cold shallow asthenosphere beneath the ERRTI highlights the potential of low thermal ridge settings to enhance the separation of mantle components. This thermally driven phenomenon results from heat preferentially diffusing into the first melting lithology, the low-solidus component. Two critical constraints emerge: (i) low-solidus, first-melting components melt preferentially, limiting the extent of melting of the higher solidus lithologies they are in contact with; (ii) the total amount of available heat, the relative solidus position and amount of each lithology collectively define the lower fertility limit of the lithology capable of melting locally (*10*, *24*, *29*). The compositional variability of the ERRTI basalts reveals a mosaic of fertile/enriched source domains (E, K, and SC) interspersed with less fertile ones (NE, N, and PL). Trace element data show that the most depleted endmembers correspond to N- and PL-MORBs, characterized by high ^176^Hf/^177^Hf and high ^143^Nd/^144^Nd, indicative of long-term evolutionary processes. The PL reservoir, in particular, appears consistent with a >1-Ga recycled lower oceanic crust entrained in mantle convection. Notably, no domains more refractory than DMM (parent to N-MORB) are observed at the ERRTI. These domains are possibly too refractory to melt under the local thermal conditions; they are usually defined as ultradepleted mantle components (*3*, *11*, *18*, *70*) and are typically associated with highly REE depleted melts, enriched in radiogenic Hf and exhibiting variable Nd isotopic ratios (*11*, *17*, *18*). While rarely detected in MORBs, they have been described in melt inclusions and, mostly, in clinopyroxenes from residual abyssal peridotites (*8*–*10*, *18*, *71*). Paradoxically, ancient ultradepleted mantle domains bearing enriched isotopic signatures have been reported along the SWIR (*72*). These parcels have been interpreted as representative of eroded slices of cratonic mantle that underwent long-term metasomatism before being entrapped into the suboceanic asthenosphere (*72*).

Highly radiogenic Hf and Nd isotopic values are also present in K- and E-MORB trends (Fig. 5D). However, direct mixing of K- and E-melts with UDM-derived melts is unlikely, as the cold ERRTI region should preclude melting of UDM domains. Instead, these isotopic signatures may arise from melt/rock reactions: either directly, by interaction with UDM domains during melt percolation/extraction, or indirectly, through the formation of secondary pyroxenites by reaction between melts and UDM domains, subsequently generating isotopically hybrid melts along the decompressional path.

The occurrence of the enriched components appears confined to the colder ridge regions near the ERRTI. Numerical models presented in the previous paragraphs, assuming homogeneously distributed low-solidus lithologies along the ridge axis, suggest that the central ridge region, where melting reaches its maximum (Fig. 7), generates melts with a limited fraction of pyroxenitic derived melts (<30%), decreasing to <20% with slightly higher mantle potential temperatures (fig. S5). This aligns with the compositional fluctuations observed in the N- and PL-MORBs, which are consistent with mixing of <25% pyroxenitic derived melts with compositions of E- and K-MORB endmembers. A major limit of our model is to consider only binary source mixing, which excludes the possibility of multiple low-solidus components contributing variably. Nevertheless, a general indication can be inferred, i.e., the total amount of low-solidus lithologies is overall low (<10%). These lithologies are partially melted together with or independently of the DMM depending on the local PT decompressional path (Figs. 6A and 7). At the segment center, mantle decompression follows hot PT paths, with large melt production that facilitates complete mixing of melts into the N-MORB matrix. By contrast, cold PT paths close to the ERRTI only allow melt production from low-solidus components, preventing homogenization and mixing with the host DMM thus revealing the source lithological variability. This is indirectly supported by the observation that the average of all measured values aligns with the regional trend (Fig. 1), confirming the multicolored composition of N-MORBs.

A clear attribution of the origin of the low-solidus components is beyond the scope of this study and requires considering the whole spectrum of incompatible elements. However, initial observations suggest that the three enriched NE-, E-, and K-components match known OIB reservoirs attributed to recycling of subduction-modified, metamorphosed oceanic crust with or without sediment contribution (*2*, *21*, *65*). Similarly, the depleted PL component may represent recycled delaminated gabbroics from the lower oceanic crust depleted by partial melting during subduction or other events. Together, these components may originate from an ancient sedimented oceanic crust finely dismembered and scattered in the sub ERRTI mantle during global mantle flow. An alternative hypothesis is their generation by stirring plume-derived filaments in the upper mantle, i.e., after sinking of a composite subducted oceanic plate deeply into the mantle and incorporation in a rising hot plume (*73*, *74*). Both scenarios require prolonged delamination and stirring within the mantle over geological timescales.

This study provides a foundation for future petrological and geochemical investigations into the structure and composition of mantle heterogeneities in “normal” mantle settings, unaffected by plume activity, irrespective of which hypothesis ultimately proves correct.

Low subridge thermal settings are not unique to RTIs but characterize large portions of ultraslow spreading ridges. Accordingly, K_2_O-rich and isotopically enriched alkalic MORBs have been reported along the Southwest Indian and Arctic ridges, attributed to the preferential extraction of low-solidus components (*75*, *76*). These findings, along with the results presented here, highlight the distinctive capacity of cold ridge environments to expose mantle heterogeneities that are typically concealed by extensive melting and mixing processes.

## MATERIALS AND METHODS

### 3D mantle thermal model

The creation of lithosphere along MOR segments involves a complex interplay of processes closely tied to the thermal structure beneath MORs, which is determined by plate movements and discontinuities along the ridge axis, such as transform faults. This study applies the numerical model developed in (*34*, *46*) to investigate the relationships between mantle temperature, melting dynamics, crustal thickness, and the geochemical characteristics of the oceanic crust in the eastern part of the Romanche transform fault. Detailed descriptions of the model methodology are available in the works of Ligi *et al.* and Bonatti *et al.* (*34*, *46*). To reduce boundary effects in the region of interest, the modeling domain was extended to include a section of the equatorial Atlantic spanning latitudes 4.6°S to 4.6°N and longitudes 40.4°W to 3.6°W. Plate boundaries were defined using a combination of global free-air gravity anomaly data, multibeam bathymetry data from our own, and the GEBCO 2024 global grid datasets (Fig. 2A). The initial temperature distribution, before the onset of melting, was derived by solving the steady-state advection-diffusion equation, as explained in the subsequent sections.

Mantle flow velocities were estimated on the basis of steady-state passive flow dynamics, using the plate-thickening model from (*46*). Corner flow, induced by seafloor spreading, was modeled in a computational domain measuring 4096 km by 1024 km horizontally and extending to a depth of 150 km. The model used a grid spacing of 1 km by 1 km by 1 km, assuming an incompressible, homogeneous, and isoviscous mantle beneath the current geometry of the equatorial MAR. Variations in plate velocity associated with distance from the Euler pole were incorporated by allowing for deviations from the rigid plate assumption. Velocity values were calculated for each point using the Euler vector for the South American and Nubian plates, derived from the MORVEL global plate motion model (*77*). Along the equatorial MAR axis, the Nubian plate’s velocity relative to South America ranged from ~30.5 mm/year at an azimuth of 76.5°N in the southern portion of the domain to ~27.5 mm/year at an azimuth of 85.5°N in the northernmost region. Mantle flow was computed using a Fourier pseudo-spectral method (*46*), assuming that the base of the rigid lithosphere corresponds to the 750°C isotherm. This isotherm was iteratively determined by solving the mantle temperature field, beginning with a constant thickness plate flow (fig. S3). Flow patterns derived from the model are shown in fig. S3. Flow patterns derived from the model with velocity magnitudes depicted at a depth of 60 km are shown in fig. S4.

The mantle temperature field was calculated throughout the 3D model domain using the over-relaxation upwind finite difference method described in (*78*). A variable grid spacing (2048 by 1024 by 101) was used, with the highest horizontal (*x* and *y*) resolution of 1 km focused near the plate boundaries. Along the vertical (*z*) dimension, the grid step was set to 1 km down to a depth of 65 km, gradually increasing to 3 km beyond 100 km depth. The final temperature distribution was computed with boundary conditions of 0°C at the surface and a constant temperature at the base of the model, set at a depth of 150 km. To explore variations in thermal conditions, different temperature distributions were generated by incrementally varying the base temperature from 1270° to 1370°C in 20°C steps.

### Basalt chemistry

Samples from SMARTIES and previous cruises have been analyzed during the PhD thesis of L.V. at the Modena University (*79*). Major elements in glasses were analyzed by electron probe microanalysis at the Microsonde Ouest (Université de Bretagne Occidentale, Ifremer, Institut Universitaire Européen de la Mer (IUEM), INSA Rennes, Université de Rennes1, and Université de Nantes) using an 15-kV acceleration voltage, a 10-nA beam, a 5-μm diameter, and 20-s counting times. Each analysis is the average of four spots. A number of primary mineral reference materials were used for corrections.

Trace elements have been measured by laser ablation inductively coupled plasma mass spectrometry, Thermo Fisher Scientific XSeriesII, at the Centro Interdipartimentale Grandi Strumenti University of Modena (CIGS-UNIMORE, Italy) and by Laser Ablation-High Resolution-ICP-MS Thermo Fisher Scientific Element XR at Pôle Spectrométrie Océan PSO (IUEM, UBO, Ifremer, CNRS, and IRD, Brest, France). Each run consists of 50 replicates with a 30-ms dwell time. Standard reference materials NIST612 and NIST614 and the natural reference sample BHVO2 have been used for mass correction.

For isotope analyses, 100 to 200 mg of clean basaltic glass chips was handpicked under a binocular microscope. The samples were first leached by H_2_O_2_ followed by a second leach in 2.5 N HCl. Each leaching step was performed in an ultrasonic bath for ∼1 hour with rinsing in between. Samples were dissolved with a HF + HNO_3_ solution. Pb, Sr, Hf, and Nd elements were sequentially separated from the same batch. The combined Pb-Sr chemistry was performed at the Dipartimento di Scienze Chimiche e Geologiche, University of Modena (Italy) following the (*80*) protocol. Samples were loaded on 150-μl Teflon columns filled with Sr-spec resin in 2 N HNO_3_. The cut containing Hf and Nd was eluted with about 2 ml of 2 N HNO_3_, followed by Ba elution with 7 N HNO_3_ and 2 N HNO_3_. Sr was eluted with Milli-Q and Pb with 6 N HCl. All acids used in the procedure were of suprapure grade. Hf and Nd were separated from the previous cut at Géo-Océan (UMR6538 CNRS-Ifremer-UBO-UBS, France). First, the REE fraction was separated in 2-ml Teflon columns filled with AG50W-X8 (200 to 400mesh) resin. Hf was then recovered following the two columns procedure of (*81*), which uses a first set of columns containing an anionic resin AG1-X8 (100 to 200 mesh) and a second set of columns containing a cationic resin AG50W-X8 (200 to 400 mesh). Nd was separated from the REE fraction in 2-ml Teflon columns filled with containing LN-spec resin (LaNthanides Specification) using 0.25 N HCl.

Pb and Sr isotopes were measured at CIGS-UNIMORE (Modena, Italy), and Hf isotopes at PSO, with a multicollector inductively coupled plasma mass spectrometer, Thermo Fisher Scientific NEPTUNE. Nd isotope ratios were analyzed with a multicollector thermo-ionization mass spectrometer, Thermo Fisher Scientific Triton at PSO. Chemical blanks have been measured for each set of chemistry, and the maximum values are less than 0.8 ng, 0.9 ng, 40 pg, and 0.9 ng, respectively for Nd, Sr, Hf, and Pb.

Mass fractionation corrections for Sr, Nd, and Hf isotope ratios were based on ^86^Sr/^88^Sr = 0.1194, ^146^Nd/^144^Nd = 0.7219, and ^179^Hf/^177^Hf = 0.7325. Repeated analyses yielded an ^87^Sr/^86^Sr ratio of 0.710246 ± 0.000031 for the NBS-987 Sr reference material (2σ external reproducibility, *n* = 45), a ^143^Nd/^144^Nd ratio of 0.511843 ± 0.000014 for the La Jolla (2σ external reproducibility, *n* = 20), and a ^176^Hf/^177^Hf = 0.282161 ± 0.000019 (2σ external reproducibility, *n* = 70) for the AMES (isotopically equivalent to JMC-475). Nd, Sr, and Hf isotope ratios were reported to the accepted values of 0.710248 for the NBS-987, 0.511850 (*82*) for the La Jolla, and 0.282160 for the AMES. The JNdi-1 and JMC-475 standards have been regularly measured with an average ratio of 0.512099 ± 0.000015 (2σ external reproducibility, *n* = 10) and of 0.282160 ± 0.000013 (2σ external reproducibility, *n* = 28), respectively. Four samples have been reanalyzed for the Sr, Nd, and Hf isotope composition, and duplicate values are consistent with previous results.

Pb isotope ratios were corrected for mass fractionation by thallium addition. Reproducibilities are 162, 167, and 144 parts per million (2σ, *n* = 34) for the ^206^Pb/^204^Pb, ^207^Pb/^204^Pb, and ^208^Pb/^204^Pb ratios, respectively. Measured values were corrected to ^206^Pb/^204^Pb = 16.9371, ^207^Pb/^204^Pb = 15.4913, and ^208^Pb/^204^Pb = 36.7213 (*83*).

## Supplementary Materials

### The PDF file includes:

Figs. S1 to S5 Legends for datasets S1 and S2

### Other Supplementary Material for this manuscript includes the following:

Datasets S1 and S2

## References

[1] S. R. Hart, Heterogeneous mantle domains: Signatures, genesis and mixing chronologies. Earth Planet. Sci. Lett. 90, 273–296 (1988).

[2] A. Zindler, S. R. Hart, Chemical geodynamics. Annu. Rev. Earth Planet. Sci. 14, 493–571 (1986).

[3] A. Stracke, A process-oriented approach to mantle geochemistry. Chem. Geol. 579, 120350 (2021).

[4] A. Stracke, B. Bourdon, The importance of melt extraction for tracing mantle heterogeneity. Geochim. Cosmochim. Acta 73, 218–238 (2009).

[5] J. F. Rudge, J. Maclennan, A. Stracke, The geochemical consequences of mixing melts from a heterogeneous mantle. Geochim. Cosmochim. Acta 114, 112–143 (2013).

[6] V. J. M. Salters, H. J. B. Dick, Mineralogy of the mid-ocean-ridge basalt source from neodymium isotopic composition of abyssal peridotites. Nature 418, 68–72 (2002). 12097907 10.1038/nature00798

[7] C.-Z. Liu, J. E. Snow, E. Hellebrand, G. Brügmann, A. von der Handt, A. Büchl, A. W. Hofmann, Ancient, highly heterogeneous mantle beneath Gakkel ridge, Arctic Ocean. Nature 452, 311–316 (2008). 18354475 10.1038/nature06688

[8] A. Cipriani, E. Bonatti, R. W. Carlson, Nonchondritic ^142^Nd in suboceanic mantle peridotites. Geochem. Geophys. Geosyst. 12, 1–8 (2011).

[9] A. Cipriani, H. K. Brueckner, E. Bonatti, D. Brunelli, Oceanic crust generated by elusive parents: Sr and Nd isotopes in basalt-peridotite pairs from the Mid-Atlantic Ridge. Geology 32, 657–660 (2004).

[10] D. Brunelli, A. Cipriani, E. Bonatti, Thermal effects of pyroxenites on mantle melting below mid-ocean ridges. Nat. Geosci. 11, 520–525 (2018).

[11] M. Willig, A. Stracke, C. Beier, V. J. M. Salters, Constraints on mantle evolution from Ce-Nd-Hf isotope systematics. Geochim. Cosmochim. Acta 272, 36–53 (2020).

[12] S. Lambart, J. M. Koornneef, M. A. Millet, G. R. Davies, M. Cook, C. J. Lissenberg, Highly heterogeneous depleted mantle recorded in the lower oceanic crust. Nat. Geosci. 12, 482–486 (2019).

[13] A. E. Saal, S. R. Hart, N. Shimizu, E. H. Hauri, G. D. Layne, Pb isotopic variability in melt inclusions from oceanic island basalts, polynesia. Science 282, 1481–1484 (1998). 9822377 10.1126/science.282.5393.1481

[14] A. V. Sobolev, A. W. Hofmann, K. P. Jochum, D. V. Kuzmin, B. Stoll, A young source for the Hawaiian plume. Nature 476, 434–437 (2011). 21832996 10.1038/nature10321

[15] J. M. Koornneef, I. Nikogosian, M. J. van Bergen, R. Smeets, C. Bouman, G. R. Davies, TIMS analysis of Sr and Nd isotopes in melt inclusions from Italian potassium-rich lavas using prototype 10^13^ Ω amplifiers. Chem. Geol. 397, 14–23 (2015).

[16] A. A. Reinhard, M. G. Jackson, J. Harvey, C. R. Brown, J. M. Koornneef, Extreme differences in ^87^Sr/^86^Sr between Samoan lavas and the magmatic olivines they host: Evidence for highly heterogeneous ^87^Sr/^86^Sr in the magmatic plumbing system sourcing a single lava. Chem. Geol. 439, 120–131 (2016).

[17] A. Stracke, F. Genske, J. Berndt, J. M. Koornneef, Ubiquitous ultra-depleted domains in Earth’s mantle. Nat. Geosci. 12, 851–855 (2019).

[18] A. Sanfilippo, V. J. M. Salters, S. Y. Sokolov, A. A. Peyve, A. Stracke, Ancient refractory asthenosphere revealed by mantle re-melting at the Arctic Mid Atlantic Ridge. Earth Planet. Sci. Lett. 566, 116981 (2021).

[19] J. Agranier, D. Blicherttoft, V. Graham, P. Debaille, F. Schiano, Albarede, The spectra of isotopic heterogeneities along the mid-Atlantic Ridge. Earth Planet. Sci. Lett. 238, 96–109 (2005).

[20] J.-G. G. Schilling, B. B. Hanan, B. McCully, R. H. Kingsley, D. Fontignie, Influence of the Sierra Leone mantle plume on the equatorial Mid-Atlantic Ridge: A Nd-Sr-Pb isotopic study. J. Geophys. Res. Solid Earth 99, 12005–12028 (1994).

[21] W. M. White, Sources of oceanic basalts: Radiogenic isotopic evidence. Geology 13, 115–118 (1985).

[22] K. E. Donnelly, S. L. Goldstein, C. H. Langmuir, M. Spiegelman, Origin of enriched ocean ridge basalts and implications for mantle dynamics. Earth Planet. Sci. Lett. 226, 347–366 (2004).

[23] C. Hémond, A. W. Hofmann, I. Vlastélic, F. Nauret, Origin of MORB enrichment and relative trace element compatibilities along the Mid-Atlantic Ridge between 10° and 24°N. Geochem. Geophys. Geosyst. 7, Q12010 (2006).

[24] J. Phipps Morgan, Thermodynamics of pressure release melting of a veined plum pudding mantle. Geochem. Geophys. Geosyst. 2, 2000GC000049 (2001).

[25] K. M. Haase, C. W. Devey, D. F. Mertz, P. Stoffers, D. Garbe-Schönberg, Geochemistry of lavas from Mohns ridge, Norwegian-Greenland Sea: Implications for melting conditions and magma sources near Jan Mayen. Contrib. Mineral. Petrol. 123, 223–237 (1996).

[26] O. Shorttle, J. Maclennan, Compositional trends of Icelandic basalts: Implications for short-length scale lithological heterogeneity in mantle plumes. Geochem. Geophys. Geosyst. 12, Q11008 (2011).

[27] N. H. Sleep, Tapping of magmas from ubiquitous mantle heterogeneities: An alternative to mantle plumes? J. Geophys. Res. 89, 10029–10041 (1984).

[28] R. F. Katz, J. F. Rudge, The energetics of melting fertile heterogeneities within the depleted mantle. Geochem. Geophys. Geosyst. 12, Q0AC16 (2011).

[29] S. Lambart, M. B. Baker, E. M. Stolper, The role of pyroxenite in basalt genesis: Melt-PX, a melting parameterization for mantle pyroxenites between 0.9 and 5 GPa. J. Geophys. Res. Solid Earth 121, 5708–5735 (2016).

[30] J.-G. G. Schilling, C. Ruppel, A. N. N. Davis, B. McCully, S. A. A. Tighe, R. H. Kingsley, J. Lin, Thermal structure of the mantle beneath the equatorial Mid-Atlantic Ridge: Inferences from the spatial variation of dredged basalt glass compositions. J. Geophys. Res. 100, 10057–10076 (1995).

[31] E. Bonatti, D. Brunelli, P. Fabretti, M. Ligi, R. A. Portaro, M. Seyler, Steady-state creation of crust-free lithosphere at cold spots in mid-ocean ridges. Geology 29, 979–982 (2001).

[32] M. Ligi, E. Bonatti, L. Gasperini, A. N. B. Poliakov, Oceanic broad multifault transform plate boundaries. Geology 30, 11–14 (2002).

[33] E. Bonatti, Anomalous opening of the Equatorial Atlantic. Earth Planet. Sci. Lett. 143, 147–160 (1996).

[34] M. Ligi, E. Bonatti, A. Cipriani, L. Ottolini, Water-rich basalts at mid-ocean-ridge cold spots. Nature 434, 66–69 (2005). 15744299 10.1038/nature03264

[35] E. Bonatti, M. Seyler, N. Sushevskaya, A cold suboceanic mantle belt at the Earth’s equator. Science 261, 315–320 (1993). 17836842 10.1126/science.261.5119.315

[36] M. Le Voyer, E. E. Cottrell, K. A. Kelley, M. M. N. Brounce, E. H. E. H. Hauri, The effect of primary versus secondary processes on the volatile content of MORB glasses: An example from the equatorial Mid-Atlantic Ridge (5°N–3°S). J. Geophys. Res. Solid Earth 120, 125–144 (2015).

[37] M. Maia, D. Brunelli, M. Ligi, SMARTIES Oceanographic cruise, RV Pourquoi PAS? (2020); 10.17600/18001107.

[38] L. Gasperini, E. Bonatti, D. Brunelli, G. Carrara, A. Cipriani, P. Fabretti, D. Gilod, M. Ligi, A. Peyve, S. Skolotnev, S. Susini, P. Tartarotti, N. Turko, New data on the geology of the Romanche F.Z., equatorial Atlantic: PRIMAR-96 cruise report. Giornale di Geologia 59, (1997).

[39] E. Bonatti, M. Ligi, L. Gasperini, A. Peyve, Y. Raznitsin, Y. J. Chen, Transform migration and vertical tectonics at the Romanche fracture zone, equatorial Atlantic. J. Geophys. Res. Solid Earth 99, 21779–21802 (1994).

[40] E. Bonatti, Y. Raznitsin, G. Bortoluzzi, F. Boudillon, G. De Alteriis, L. Gasperini, M. Gasperini, G. Giaquinto, M. Ligi, M. Sacchi, S. Skolotnev, V. Trofimov, N. Turco, M. Zacharov, J.-M. Auzende, V. Mamaloukis Frangoulis, R. Searle, Geological studies of the eastern part of the Romanche transform (equatorial Atlantic): A first report. Giornale di geologia 53, 31–48 (1991).

[41] W. F. McDonough, S. s Sun, The composition of the Earth. Chem. Geol. 120, 223–253 (1995).

[42] C. M. Meyzen, J. N. Ludden, E. Humler, B. Luais, M. J. Toplis, C. Mével, M. Storey, New insights into the origin and distribution of the DUPAL isotope anomaly in the Indian Ocean mantle from MORB of the Southwest Indian Ridge. Geochem. Geophys. Geosyst. 6, Q11K11 (2005).

[43] A. Gale, C. A. Dalton, C. H. Langmuir, Y. Su, J.-G. G. Schilling, The mean composition of ocean ridge basalts. Geochem. Geophys. Geosyst. 14, 489–518 (2013).

[44] A. Ewart, “The mineralogy and petrology of tertiary-recent orogenic volcanic rocks; with special reference to the andesitic-basaltic compositional range” in Andesites: Orogenic andesites and related rocks. R.S. Thorpe, Ed., (Wiley, Chichester, 1982) pp. 26–87.

[45] P. J. Fox, D. G. Gallo, A tectonic model for ridge-transform-ridge plate boundaries: Implications for the structure of oceanic lithosphere. Tectonophysics 104, 205–242 (1984).

[46] M. Ligi, M. Cuffaro, F. Chierici, A. Calafato, Three-dimensional passive mantle flow beneath mid-ocean ridges: An analytical approach. Geophys. J. Int. 175, 783–805 (2008).

[47] M. Pertermann, M. M. Hirschmann, Partial melting experiments on a MORB-like pyroxenite between 2 and 3 GPa: Constraints on the presence of pyroxenite in basalt source regions from solidus location and melting rate. J. Geophys. Res. Solid Earth 108, 2125 (2003).

[48] S. Lambart, D. Laporte, P. Schiano, Markers of the pyroxenite contribution in the major-element compositions of oceanic basalts: Review of the experimental constraints. Lithos 160-161, 14–36 (2013).

[49] N. Harmon, C. A. Rychert, J. M. Kendall, M. Agius, P. Bogiatzis, S. Tharimena, Evolution of the oceanic lithosphere in the equatorial Atlantic from Rayleigh wave tomography, evidence for small-scale convection from the PI-LAB experiment. Geochem. Geophys. Geosyst. 21, e2020GC009174 (2020).

[50] C. Herzberg, P. D. Asimow, N. Arndt, Y. Niu, C. M. Lesher, J. G. Fitton, M. J. Cheadle, A. D. Saunders, Temperatures in ambient mantle and plumes: Constraints from basalts, picrites, and komatiites. Geochem. Geophys. Geosyst. 8, Q02006 (2007).

[51] A. Cipriani, E. Bonatti, D. Brunelli, M. Ligi, 26 Million years of mantle upwelling below a segment of the Mid Atlantic Ridge: The Vema lithospheric section revisited. Earth Planet. Sci. Lett. 285, 87–95 (2009).

[52] P. M. Gregg, J. Lin, M. D. Behn, L. G. J. Montési, Spreading rate dependence of gravity anomalies along oceanic transform faults. Nature 448, 183–187 (2007). 17625563 10.1038/nature05962

[53] I. Grevemeyer, L. H. Rüpke, J. P. Morgan, K. Iyer, C. W. Devey, Extensional tectonics and two-stage crustal accretion at oceanic transform faults. Nature 591, 402–407 (2021). 33731945 10.1038/s41586-021-03278-9

[54] M. M. Hirschmann, T. Kogiso, M. B. Baker, E. M. Stolper, Alkalic magmas generated by partial melting of garnet pyroxenite. Geology 31, 481 (2003).

[55] Y. Liang, Trace element fractionation and isotope ratio variation during melting of a spatially distributed and lithologically heterogeneous mantle. Earth Planet. Sci. Lett. 552, 116594 (2020).

[56] J. M. Koornneef, A. Stracke, B. Bourdon, M. A. Meier, K. P. Jochum, B. Stoll, K. Grönvold, Melting of a two-component source beneath Iceland. J. Petrol. 53, 127–157 (2012).

[57] A. Stracke, A. W. Hofmann, S. R. Hart, FOZO, HIMU, and the rest of the mantle zoo. Geochem. Geophys. Geosyst. 6, (2005).

[58] S. R. Hart, E. H. Hauri, L. A. Oschmann, J. A. Whitehead, Mantle plumes and entrainment: Isotopic evidence. Science 256, 517–520 (1992). 17787949 10.1126/science.256.5056.517

[59] B. Mougel, A. Agranier, C. Hemond, P. Gente, A highly unradiogenic lead isotopic signature revealed by volcanic rocks from the East Pacific Rise. Nat. Commun. 5, 4474 (2014). 25027032 10.1038/ncomms5474

[60] M. Tang, W. F. McDonough, R. D. Ash, Europium and strontium anomalies in the MORB source mantle. Geochim. Cosmochim. Acta 197, 132–141 (2017).

[61] A. E. Saal, M. D. Kurz, S. R. Hart, J. S. Blusztajn, J. Blichert-Toft, Y. Liang, D. J. Geist, The role of lithospheric gabbros on the composition of Galapagos lavas. Earth Planet. Sci. Lett. 257, 391–406 (2007).

[62] L. V. Danyushevsky, M. R. Perfit, S. M. Eggins, T. J. Falloon, Crustal origin for coupled “ultra-depleted” and “plagioclase” signatures in MORB olivine-hosted melt inclusions: Evidence from the Siqueiros Transform Fault, East Pacific Rise. Contrib. Mineral. Petrol. 144, 619–637 (2003).

[63] A. V. Sobolev, A. W. Hofmann, I. K. Nikogosian, Recycled oceanic crust observed in ‘ghost plagioclase’ within the source of Mauna Loa lavas. Nature 404, 986–990 (2000). 10801125 10.1038/35010098

[64] R. Tilhac, K. Hidas, B. Oliveira, C. J. Garrido, Evidence of ghost plagioclase signature induced by kinetic fractionation of europium in the Earth’s mantle. Nat. Commun. 14, 1099 (2023). 36841809 10.1038/s41467-023-36753-0 PMC9968321

[65] A. Stracke, M. Bizimis, V. J. M. Salters, Recycling oceanic crust: Quantitative constraints. Geochem. Geophys. Geosyst. 4, 8003 (2003).

[66] G. M. Nowell, D. G. Pearson, D. R. Bell, R. W. Carlson, C. B. Smith, P. D. Kempton, S. R. Noble, Hf isotope systematics of kimberlites and their megacrysts: New constraints on their source regions. J. Petrol. 45, 1583–1612 (2004).

[67] A. Fitzpayne, A. Giuliani, G. H. Howarth, B. J. Peters, M. A. Fehr, R. Maas, Major-, trace-element and Sr-Nd-Hf isotope geochemistry of diamondiferous dykes from Tonguma and Koidu, Sierra Leone: Highly micaceous kimberlites formed by assimilation of metasomatised lithospheric mantle rocks. Chem. Geol. 630, 121475 (2023).

[68] E. Bonatti, M. Ligi, A. M. Borsetti, L. Gasperini, A. Negri, R. Sartori, Lower cretaceous deposits trapped near the equatorial Mid-Atlantic ridge. Nature 380, 518–520 (1996).

[69] E. Bonatti, K. Crane, Oscillatory spreading explanation of anomalously old uplifted crust near oceanic transforms. Nature 300, 343–345 (1982).

[70] V. J. M. Salters, S. Mallick, S. R. Hart, C. E. Langmuir, A. Stracke, Domains of depleted mantle: New evidence from hafnium and neodymium isotopes. Geochem. Geophys. Geosyst. 12, Q08001 (2011).

[71] A. Stracke, J. E. Snow, E. Hellebrand, A. Von der Handt, B. Bourdon, K. Birbaum, D. Günther, Abyssal peridotite Hf isotopes identify extreme mantle depletion. Earth Planet. Sci. Lett. 308, 359–368 (2011).

[72] C.-Z. Liu, H. J. B. Dick, R. N. Mitchell, W. Wei, Z.-Y. Zhang, A. W. Hofmann, J.-F. Yang, Y. Li, Archean cratonic mantle recycled at a mid-ocean ridge. Sci. Adv. 8, eabn6749 (2022). 35648865 10.1126/sciadv.abn6749 PMC9159695

[73] J. Phipps Morgan, W. J. Morgan, Two-stage melting and the geochemical evolution of the mantle: A recipe for mantle plum-pudding. Earth Planet. Sci. Lett. 170, 215–239 (1999).

[74] A. Soltanmohammadi, M. Grégoire, F. J. Fontaine, L. P. Bédard, M. Blanchard, M. Rabinowicz, Melt percolation, concentration and Dyking in the Hawaiian mantle plume and overriding lithosphere: Links to the evolution of lava composition along the volcanic chain. J. Petrol. 63, 1–24 (2022).

[75] J. J. Standish, H. J. B. Dick, P. J. Michael, W. G. Melson, T. O’Hearn, MORB generation beneath the ultraslow spreading Southwest Indian Ridge (9–25°E): Major element chemistry and the importance of process versus source. Geochem. Geophys. Geosyst. 9, Q05004 (2008).

[76] F. Nauret, J. E. Snow, E. Hellebrand, D. Weis, Geochemical composition of K-rich lavas from the lena trough (Arctic Ocean). J. Petrol. 52, 1185–1206 (2011).

[77] C. DeMets, R. G. Gordon, D. F. Argus, Geologically current plate motions. Geophys. J. Int. 181, 1–80 (2010).

[78] J. P. Morgan, D. W. Forsyth, Three-dimensional flow and temperature perturbations due to a transform offset: Effects on oceanic crustal and upper mantle structure. J. Geophys. Res. 93, 2955, 2966 (1988).

[79] L. Verhoest, “Melting a heterogeneous Earth mantle under an extreme thermal gradient,” thesis, University of Modena and Reggio Emilia, Modena (2022).

[80] C. Deniel, C. Pin, Single-stage method for the simultaneous isolation of lead and strontium from silicate samples for isotopic measurements. Anal. Chim. Acta 426, 95–103 (2001).

[81] J. Blichert-Toft, C. Chauvel, F. Albarède, Separation of Hf and Lu for high-precision isotope analysis of rock samples by magnetic sector-multiple collector ICP-MS. Contrib. Mineral. Petrol. 127, 248–260 (1997).

[82] W. Zhang, Z. Hu, Estimation of isotopic reference values for pure materials and geological reference materials. At. Spectrosc. 41, 93–102 (2020).

[83] E. J. Catanzaro, T. J. Murphy, W. R. Shields, E. L. Garner, Absolute isotopic abundance ratios of common, equal-atom, and radiogenic lead isotopic standards. J. Res. Natl. Bur. Stand A Phys. Chem. 72A, 261 (1968). 31824095 10.6028/jres.072A.025 PMC6624684

[84] S. Lambart, D. Laporte, P. Schiano, An experimental study of pyroxenite partial melts at 1 and 1.5GPa: Implications for the major-element composition of Mid-Ocean Ridge Basalts. Earth Planet. Sci. Lett. 288, 335–347 (2009).

