# Supplementary material for: Large melt diversity at a mid-ocean ridge thermal low
Source: Sci Adv. 2025 Apr 30;11(18):eadv4654. doi: 10.1126/sciadv.adv4654 (PMC12042885; doi:10.1126/sciadv.adv4654)
Supplement: Supplementary file 1 — Figs. S1 to S5 Legends for datasets S1 and S2 [file sciadv.adv4654_sm.pdf]

Science Advances
AAAS

Supplementary Materials for

Large melt diversity at a mid-ocean ridge thermal low

Daniele Brunelli *et al.*

Corresponding author: Daniele Brunelli, daniele.brunelli@unimore.it

Sci. Adv. **11**, eadv4654 (2025)
DOI: 10.1126/sciadv.adv4654

The PDF file includes:

Figs. S1 to S5
Legends for datasets S1 and S2

Other Supplementary Material for this manuscript includes the following:

Datasets S1 and S2

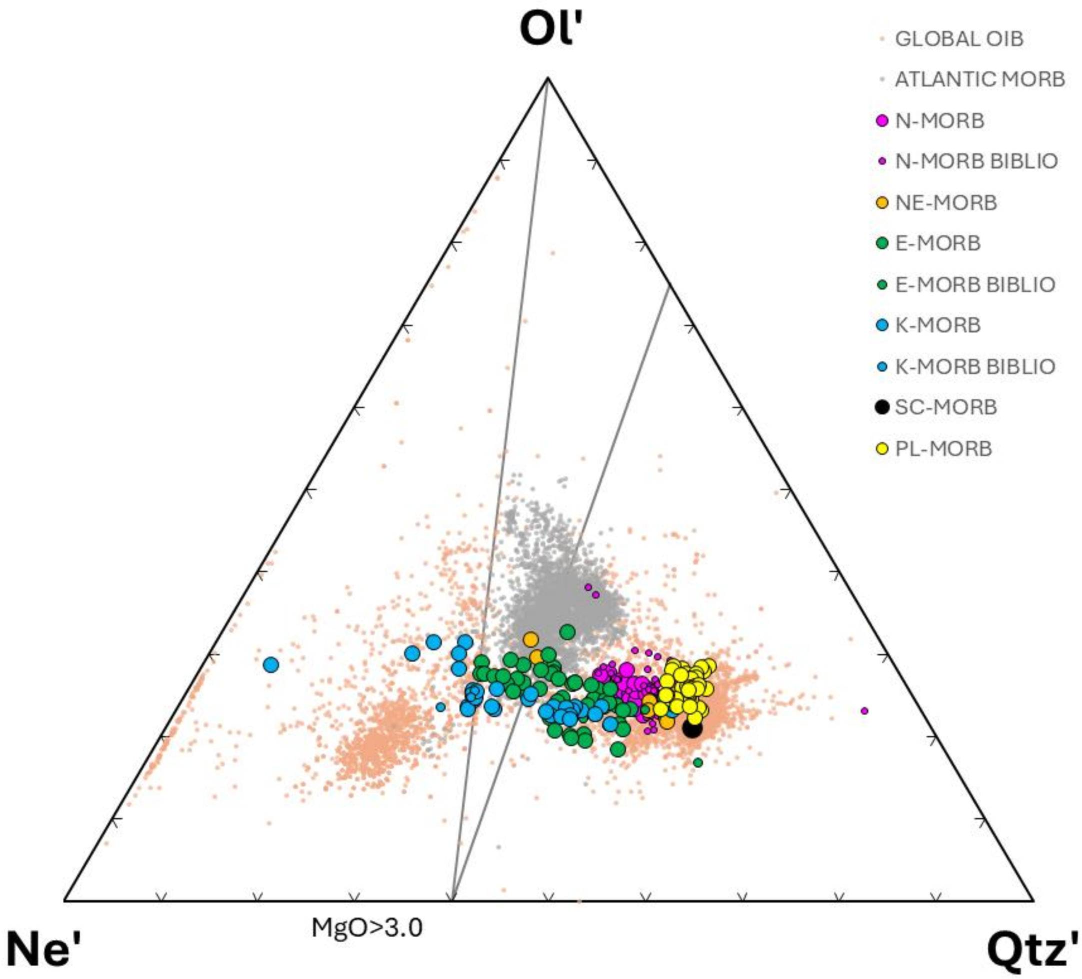

Fig. S1.

Normative composition of the basaltic glasses from the Romanche-Chain ridge segment. Large symbols are basaltic glasses from this work, small symbols are published data from (*20*, *30*, *36*). Several samples from the K-MORB group are nepheline normative. The overall compositional trend does not match the Atlantic MORB field (grey small circles) but superposes partially with the global OIB field (orange small circles) in the silica enriched terms and trend in between MORBs and OIB toward ne-normative terms. Large symbols are data from this work, small symbols are literature data color-coded according to our compositional grouping.

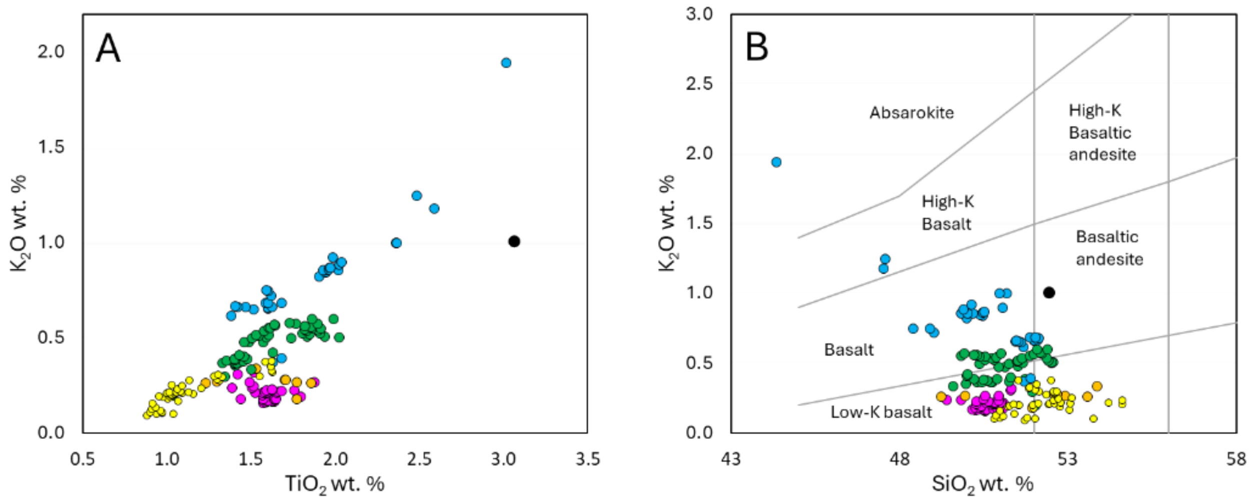

Fig. S2.

Compositions of the basaltic glasses from the Romanche-Chain ridge segment. (A) K_2O vs. TiO_2. Samples are color coded as in Fig. S1. The K-MORB group shows unique potassium enrichment as high as 2.0 weight %, a unique feature in MORB glasses far from hotspots. **(B)** K_2O vs. SiO_2. Compositional fields are from (*44*) showing that K-MORBs reach the compositional characteristics of high-K basalt up to absarokite.

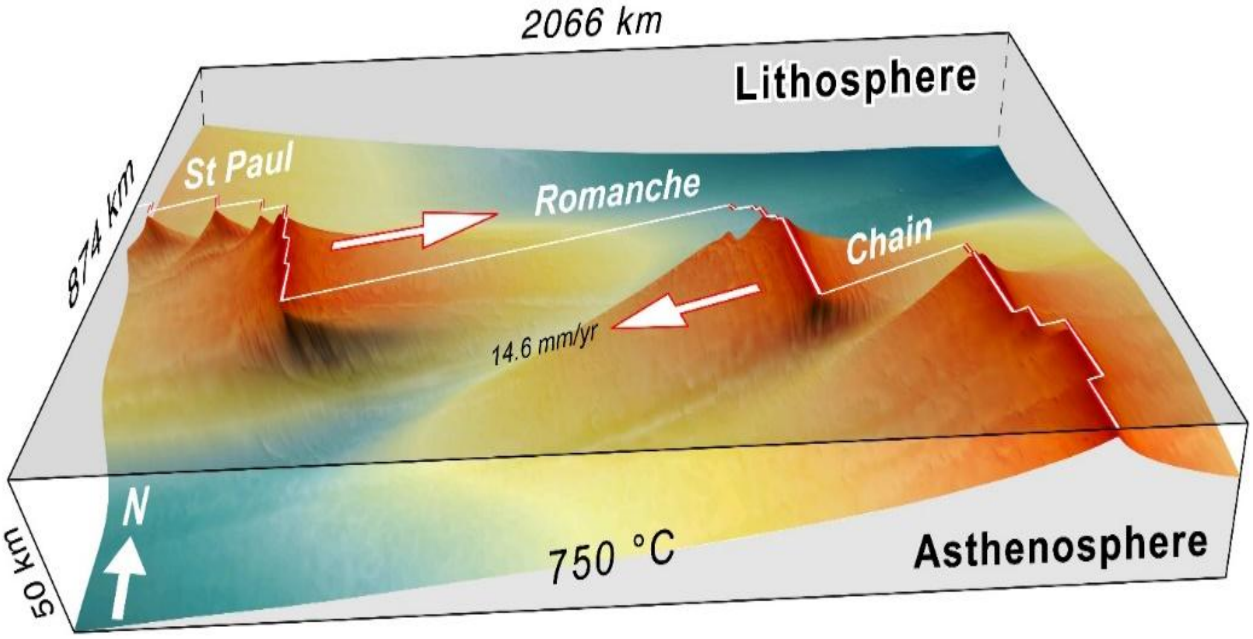

Fig. S3.

Details of plate boundary geometry and boundary conditions used in the mantle flow model. The assumed plate boundary geometry between the Nubian and South American plates mimics the equatorial pattern of the Mid-Atlantic Ridge. In this model, mantle flow is treated as isoviscous and incompressible, occurring in a half-space driven by the divergent motion of rigid plates at a velocity of 29.2 mm/yr. The depth of the 750 °C isotherm is used to approximate the base of the rigid plates, forming the upper boundary of the passive mantle flow model. This model simulates the progressive thickening of plates away from the ridge axis and was developed by iteratively solving the mantle temperature field, using a thin-plate flow model as the starting condition.

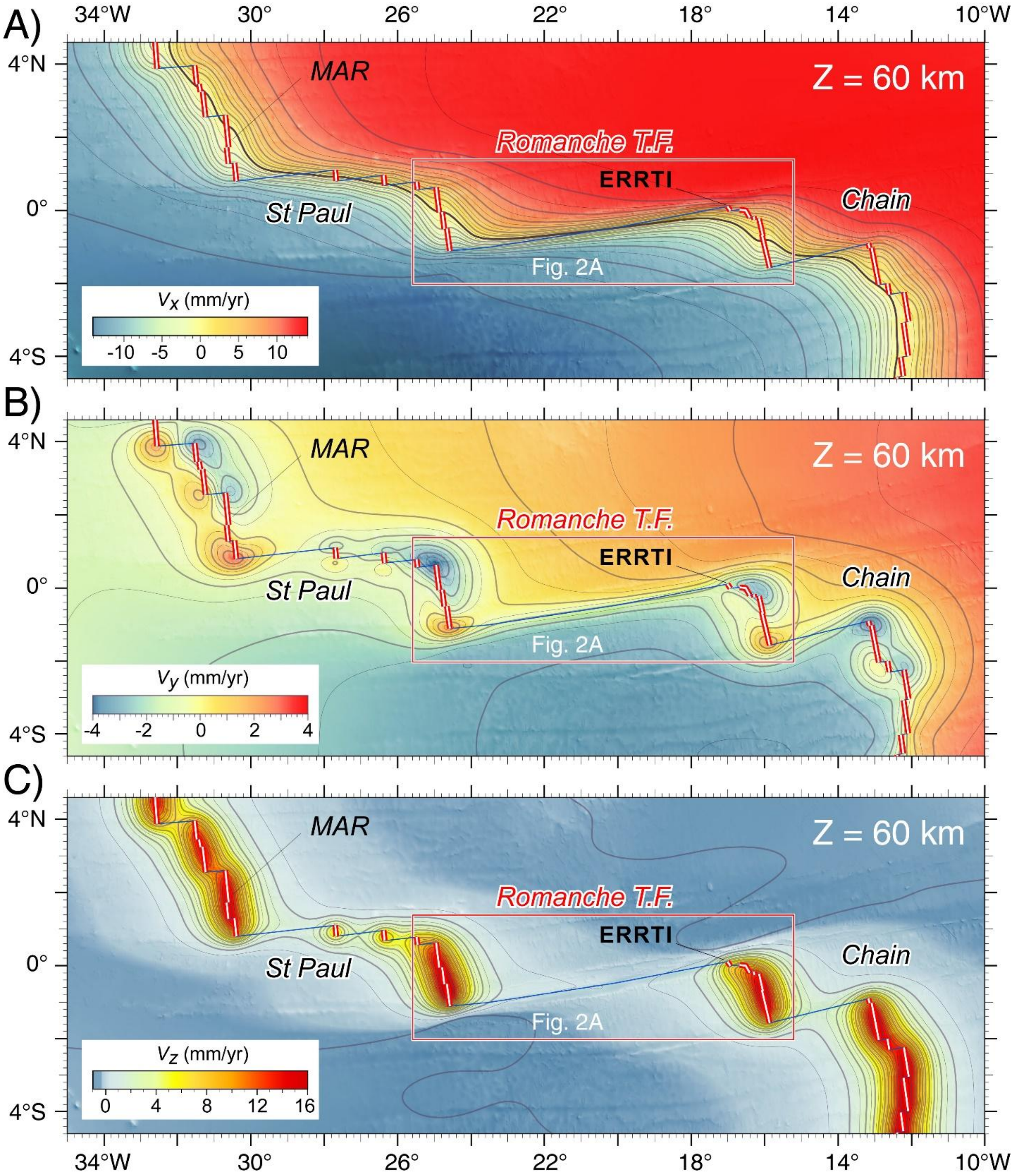

Fig. S4.

Mantle flow velocity components for the thickening-plate model at a depth of 60 km. The colored grids and contours depict the predicted velocity magnitude: (**A**) across-axis velocities (V_x) with contours at 1 mm/yr intervals, (**B**) along-axis velocities (V_y), and (**C**) vertical velocities (V_z) contoured at 0.5 mm/yr intervals.

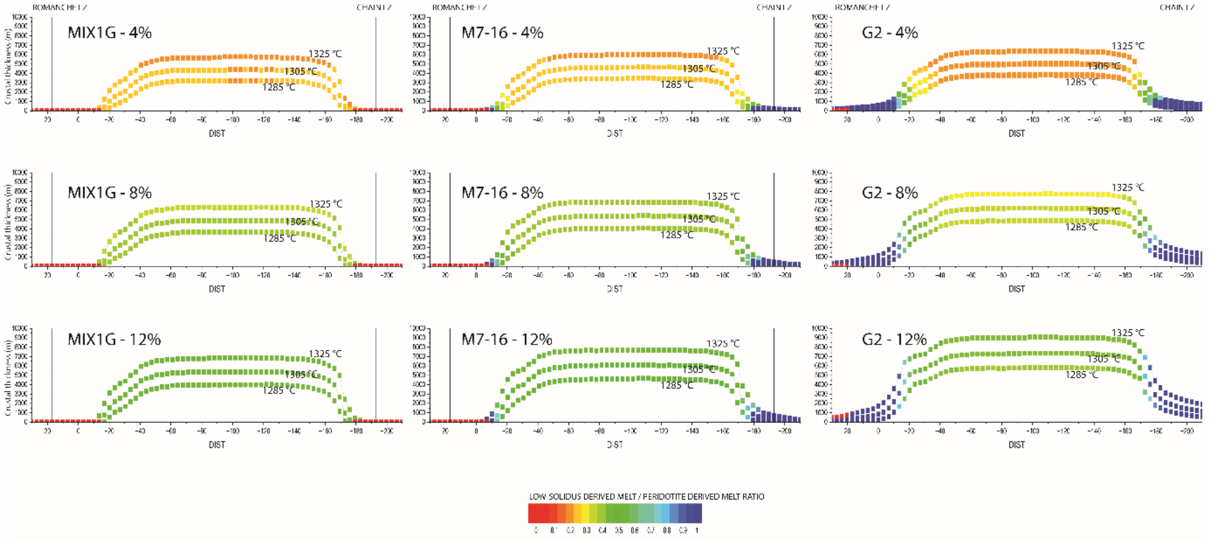

Fig. S5. Predicted crustal thickness and relative melt composition. MeltPX estimated crustal thickness along the Romanche-Chain ridge segment calculated at mantle T_p of 1285, 1305 and 1325 °C with variable amount of low solidus component dispersed in the DM peridotite (4-8-12 wt.%). Every curve is color-coded according to the relative amount of low-solidus component in the final melt mixture ranging from 0 = no low-solidus component (only DM-derived melt) to 1 = pure low-solidus component. The scale bar shows the volume ratio between the two components. The two thermal regions at both ridge ends have the potential to produce melts purely derived low-solidus pyroxenites ranging in composition from M7-16 to G2.

Supplementary Data

Data S1. (separate file)

Data S2 (separate file)
